# Different apoptotic pathways are induced from various intracellular sites by tetraphenylporphyrins and light

**DOI:** 10.1038/sj.bjc.6690014

**Published:** 1999-09-24

**Authors:** B B Noodt, K Berg, T Stokke, Q Peng, J M Nesland

**Affiliations:** Departments of Pathology and Biophysics, Institute for Cancer Research, The Norwegian Radium Hospital, Montebello, 0310 Oslo, Norway

**Keywords:** apoptosis, photodynamic therapy, intracellular localization, flow cytometry, quantification

## Abstract

The induction of apoptosis from different intracellular sites was studied by exposing V79 Chinese hamster fibroblasts to photodynamic therapy (PDT) with various porphyrins and light. The effects of two lipophilic, intracellular membrane-localized porphyrins, tetra(3-hydroxyphenyl)porphyrin (3THPP) and Photofrin, were compared with that of two sulphonated *meso* -tetraphenylporphines (TPPS_2a_ and TPPS_4_), which are taken up into lysosomes by endocytosis. Apoptotic fractions induced by the various dyes and light were quantified by flow cytometry using the terminal deoxynucleotidyl transferase (TdT) assay. Cell fragmentation was measured in parallel, while the nuclear morphology of apoptotic cells was studied by fluorescence microscopy. Different kinetics were found for the induction of DNA strand breaks characteristic of apoptotic cells. PDT-induced damage to membranes resulted in an increasing number of apoptotic cells for about 12 h after PDT. After damage tolysosomes, apoptotic cells were not detected until more than 12 h after PDT. Furthermore, apoptotic bodies were not observed after PDT-induced damage to intracellular membranes, whereas apoptosis induced from lysosomal sites was characterized by extensive cell fragmentation. Cell fragmentation occurred in combination with or in the absence of nuclear fragmentation. The results support the idea that the degradation phase of apoptosis can consist of a sequence of independent steps rather than a common final pathway. © 1999 Cancer Research Campaign


*British Journal of Cancer* (1999) **79**(1), 72–81
© 1999 Cancer Research Campaign

### Different apoptotic pathways are induced from various intracellular sites by tetraphenylporphyrins and light

**BB Noodt, K Berg, T Stokke, Q Peng and JM Nesland**

Departments of Pathology and Biophysics, Institute for Cancer Research, The Norwegian Radium Hospital, Montebello, 0310 Oslo, Norway

**Summary** The induction of apoptosis from different intracellular sites was studied by exposing V79 Chinese hamster fibroblasts to photodynamic therapy (PDT) with various porphyrins and light. The effects of two lipophilic, intracellular membrane-localized porphyrins, tetra(3-hydroxyphenyl)porphyrin (3THPP) and Photofrin, were compared with that of two sulphonated *meso*-tetraphenylporphines ($TPPS_{2a}$ and $TPPS_4$), which are taken up into lysosomes by endocytosis. Apoptotic fractions induced by the various dyes and light were quantified by flow cytometry using the terminal deoxynucleotidyl transferase (TdT) assay. Cell fragmentation was measured in parallel, while the nuclear morphology of apoptotic cells was studied by fluorescence microscopy. Different kinetics were found for the induction of DNA strand breaks characteristic of apoptotic cells. PDT-induced damage to membranes resulted in an increasing number of apoptotic cells for about 12 h after PDT. After damage to lysosomes, apoptotic cells were not detected until more than 12 h after PDT. Furthermore, apoptotic bodies were not observed after PDT-induced damage to intracellular membranes, whereas apoptosis induced from lysosomal sites was characterized by extensive cell fragmentation. Cell fragmentation occurred in combination with or in the absence of nuclear fragmentation. The results support the idea that the degradation phase of apoptosis can consist of a sequence of independent steps rather than a common final pathway.

**Keywords:** apoptosis; photodynamic therapy; intracellular localization; flow cytometry; quantification

Cell death can occur as a neatly regulated complex process called apoptosis or cell suicide. It is usually assumed that different signalling pathways for apoptosis converge into a propagation phase, followed by a degradation phase consisting of a series of common final steps. As a result, cells round up and loosen from their surroundings. Often, membrane blebbing is observed and DNA is fragmented at internucleosomal sites. Finally, the dying cells form apoptotic bodies containing cytoplasm, whole organelles and nuclear fragments (Kerr et al, 1972; Wyllie et al, 1980). Apoptosis is of importance in development and homeostasis of tissues and organisms, and occurs in response to extracellular, regulating or damaging factors. Growth factors, hormones, irradiation, heat and drugs may induce or prevent apoptosis, often in a cell-specific manner. Deregulation of a cell's ability to receive death signals or to undergo cell suicide contributes to a register of serious diseases, including cancer (Fisher, 1994). In cancer therapy, prognosis seems to be correlated with induction of apoptosis (Kerr et al, 1994; Soini et al, 1996). Drug resistance or suboptimal response to a cancer therapy may be connected to the cells' lack of ability to undergo apoptosis (Hickman, 1996).

Photodynamic therapy (PDT) is a promising type of cancer therapy based on selective retention in tumour tissue of photosensitizing dyes that have a cytotoxic effect when activated by light. The dyes are taken up into cells by different pathways because of their molecular structure, charge and solubility (Henderson and Dougherty, 1992; Berg, 1996). They act mainly via singlet oxygen (Weishaupt et al, 1976) when activated by light of suitable wavelengths and damage biomolecules only in their immediate proximity (< 0.02 μm) (Moan and Berg, 1991). In recent years, it has been shown that PDT induces cell death by apoptosis in a cell-specific and dye-dependent manner (Agarwal et al, 1991; Zaidi et al, 1993; He et al, 1994; Luo et al, 1996; Noodt et al, 1996; Webber et al, 1996; Zhou et al, 1996; Luo and Kessel, 1997). The dye dependency might be related to the subcellular localization of the dyes for activating triggers of apoptosis. For further investigation of apoptosis induced by PDT, we have chosen the lipophilic Photofrin, which is used in the clinic, and three tetraphenylporphyrins that differ only in side groups on their phenyl rings. Thus, they are increasingly lipophilic in the order $TPPS_4$–$TPPS_{2a}$–3THPP. The intracellular localization of these dyes has been studied earlier (Berg et al, 1990; Noodt et al, 1993). 3THPP and Photofrin are taken up into intracellular membranes, and Photofrin has been shown to target mitochondrial membranes as well as the endoplasmic reticulum and the Golgi system. The two other dyes are taken up initially into lysosomes via endocytosis. In the present study, apoptosis was quantified flow cytometrically by the terminal deoxynucleotidyl transferase (TdT) assay at different time intervals after PDT. The results indicate that different apoptotic pathways are triggered by the various photosensitizers and light.

*Received 8 October 1997*
*Revised 11 March 1998*
*Accepted 18 March 1998*

*Correspondence to:* BB Noodt

### MATERIALS AND METHODS

### Cells

All experiments were performed on V79 Chinese hamster lung fibroblasts. The cells were cultured in monolayer in Eagle's minimal essential medium (MEM) with Hanks' salts, containing 10% fetal calf serum, 100 U $ml^{-1}$ penicillin and 100 μg $ml^{-1}$ streptavidin. The cells were kept at 37°C with 5% carbon dioxide added to the humidified air and subcultured twice a week to sustain exponential growth.

### Chemicals

Two sulphonated *meso*-tetraphenylporphines ($TPPS_4$ and $TPPS_{2a}$) and tetra(3-hydroxyphenyl)porphyrin (3THPP) were obtained from Porphyrin Products (Logan, UT, USA), while Photofrin was provided by Photofrin Medical (Raritan, NJ, USA). Stock solutions were prepared of $TPPS_4$ (90 mg ml$^{-1}$) and Photofrin (2.0 mg ml$^{-1}$) in water and of $TPPS_{2a}$ and 3THPP (0.5 mg ml$^{-1}$) in 0.1 N sodium hydroxide. The solutions were sterilized by filtration and stored at – 70°C.

### Photodynamic therapy (PDT)

In all experiments, $5 \times 10^5$ V79 cells were seeded out in 25-cm$^2$ cell culture plastic flasks (Nunc) containing 5 ml of medium and kept in the incubator for about 4 h for attachment of the cells to the substratum. The photosensitizers were then added to the medium, still containing fetal calf serum, to the following final concentrations: 3THPP (0.2 µg ml$^{-1}$), Photofrin (2.5 µg ml$^{-1}$), $TPPS_{2a}$ (0.5 µg ml$^{-1}$) and $TPPS_4$ (180 µg ml$^{-1}$). The concentrations were chosen to obtain the same level of cell death with a given light dose. After 18 h of incubation, the cells were exposed to blue light (maximum at 405 nm) from a bench with four fluorescing tubes (model 3026, Applied Photophysics, Surrey, UK) at an intensity of 11 ± 0.5 W m$^{-2}$. Immediately after light exposure, the medium was removed, fresh medium without sensitizer was added and the cells brought back to 37°C. In some experiments, the dyes were washed away from the cell surface by removing the medium and adding fresh medium without sensitizers 1 h before irradiation.

### Cell death assay

To measure total cell death induced by PDT, the cells in each flask were divided into five sectors that were irradiated with increasing light doses as described previously (Noodt et al, 1996). Twenty-six hours after PDT, when dead cells had loosened from the bottom of the culture flasks, the remaining attached cells were washed with phosphate-buffered saline (PBS), fixed with 70% ethanol and stained with methylene blue. Excess stain was washed off carefully and the absorption of 600-nm red light was measured in each sector with a spectrophotometer. The absorption of light in an empty flask treated in parallel was subtracted from all values. The absorption of light by surviving cells in each sector was divided by the absorption by the cells in the first sector which had not been exposed to light and which was set to 100% cell survival. Total cell death was defined as the percentage of cells not surviving the cell death assay. Sensitizers or light alone had no cytotoxic effect.

### Terminal deoxynucleotide transferase (TdT) assay

For detection of DNA strand breaks at different time intervals after light exposure, the PDT-treated cells were washed with cold PBS (4°C), scraped off the bottom of the culture flasks with a rubber policeman and transferred to Eppendorf tubes. Loose cells in the medium were spun down and included in each sample. Freshly prepared 1% paraformaldehyde in PBS was added for 10 min while pelleting the cells. The supernatant was discarded, the wet pellet resuspended by vortexing, and the cells finally fixed in 100% methanol for storage at –20°C.

For fluorescence staining of the DNA strand breaks by the TdT assay (Gorczyca et al, 1993), fixed and stored cells in methanol were washed with PBS. A TdT solution of 30 µl containing 0.3 µl of biotin-16-dUTP (0.5 nmol), 0.12 µl of TdT (10 units), 3 µl of reaction buffer (1 M potassium cacodylate, 125 mM Tris-HCl, 1.25 mg/ml$^{-1}$ bovine serum albumin, pH 6.6), 1.8 µl of cobalt chloride (final 1.5 mM), 0.03 µl of dithiothreitol (DTT, final 0.01 mM) and water (all chemicals from Boehringer Mannheim) was added to each sample. After incubation for 30 min at 37°C, the cells, now with elongated DNA strand breaks, were again washed, once in PBS and twice in PBS with 1% Triton X-100. Then 1 µl of streptavidin–fluorescein (1:50, Amersham) in 49 µl of water with 0.1% Triton X-100 and 2% skimmed milk powder was added for 30 min at 4°C for fluorescence staining of the elongated free DNA ends. Finally, 500 µl of PBS–Triton X-100 containing 2 µg ml$^{-1}$ Hoechst 33258 was added for fluorescence staining of total DNA. Each sample was filtered by a 50-nm filter and further analysed.

### Flow cytometry and cell sorting

The stained samples were analysed by a FACStar Plus flow cytometer (Becton Dickinson, San Jose, CA, USA). Blue fluorescence (402–446 nm) from total DNA stained by Hoechst 33258 was excited with a krypton laser operated at 50 mW in the UV (351 nm and 356 nm). Green fluorescence (515–545 nm) from fluorescein labelling the extended DNA strand breaks was excited by an argon laser tuned to 488 nm (200 mW). The intensity of both blue and green fluorescence from $10^4$ cells was measured for each sample and analysed by Lysis II software. Single cells were discriminated from doublets and larger cell aggregates by gating on the pulse width of the Hoechst 33258 signals.

For calculation of the apoptotic fraction of cells in each sample, the fluorescence representing DNA strand breaks was plotted relative to the DNA content of the cells, as described previously (Noodt et al, 1996) and as shown in Figure 1. Non-specific fluorescein background staining was defined on plots for control samples that were fluorescence stained by streptavidin–fluorescein without previous DNA strand break elongation by TdT (Figure 1A). A gate was set for quantification such that more than 98% of the control stained cells were included (regions R1 in Figure 1B). Cells that were fluorescence stained after TdT treatment (Figure 1B) and which showed an increased fluorescence intensity above the maximal background level (regions R2 in Figure 1B) were defined as apoptotic owing to an increased number of DNA strand breaks. To calculate apoptotic fractions, only cells with a DNA content of more than half that of $G_1$ cells (relative blue fluorescence intensity > 100 in Figure 1) were included. This was carried out to avoid counting more than one cell fragment derived from a single cell. The background level was subtracted from all numbers.

Cell fragmentation was quantified by dividing the number of signals from cell fragments with a DNA content of less than half that of $G_1$ cells (regions R3 in Figure 1) by the total number of signals. These should be considered only as relative numbers because fragmentation may occur to a varying extent and cell fragments can get lost during the washing procedures.

### Fluorescence microscopy

The morphology of the nuclei of apoptotic cells was studied by fluorescence microscopy, detecting the green fluorescence from fluorescein after staining of DNA strand breaks in the TdT assay. Apoptotic cells that had been sorted by the FACStar flow

© Cancer Research Campaign 1999

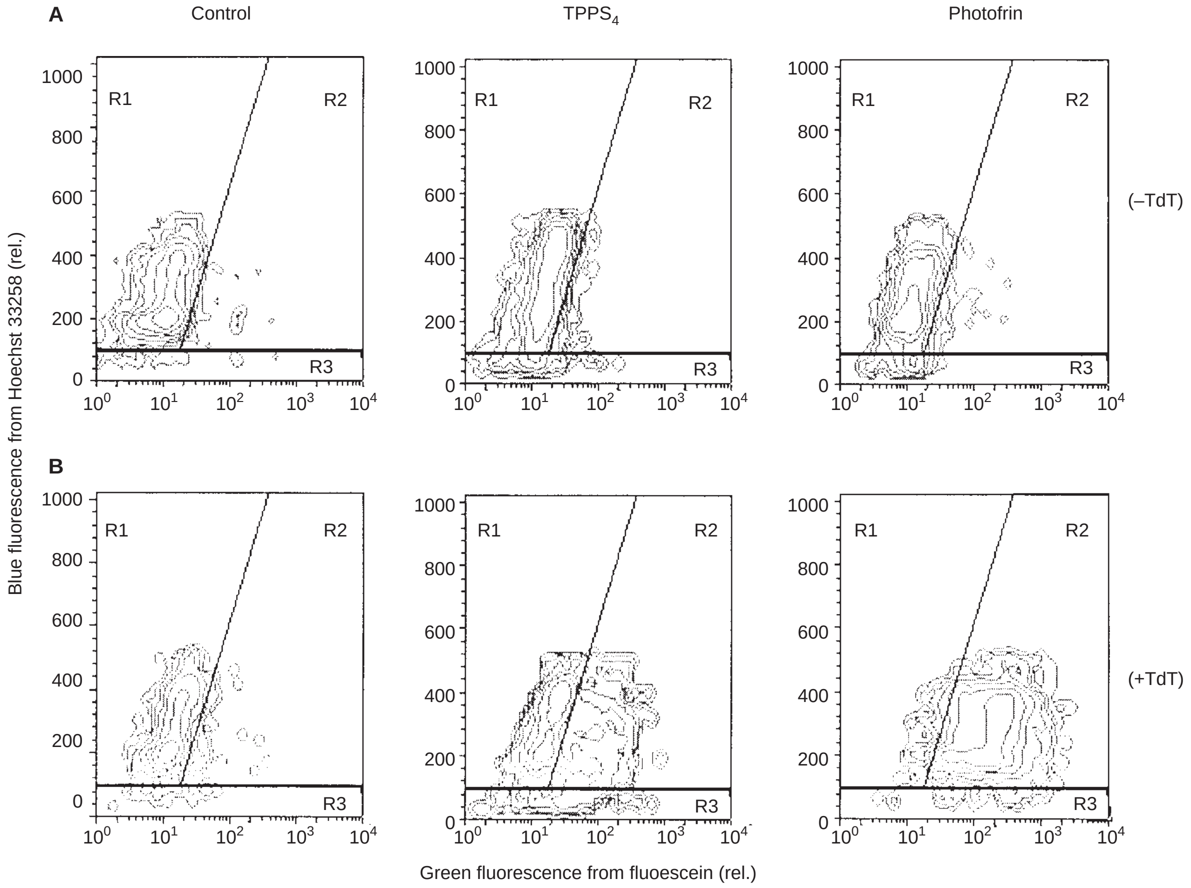

**Figure 1** Contour plots of flow cytometric data showing DNA strand breaks relative to total DNA represented by green fluorescence from fluorescein and blue fluorescence from Hoechst 33258, respectively, in V79 control cells and cells PDT-treated with $TPPS_4$ and Photofrin. Background staining (regions R1) was determined on contour plots for control samples that were fluorescein stained without previous elongation of DNA strand breaks by TdT (**A**). Samples treated with TdT (**B**) show the gating of the apoptotic cells with increased fluorescence staining of DNA strand breaks above the background and a DNA content more than half that in $G_1$ cells (regions R2). Cells with a lower DNA content than half $G_1$ (regions R3) were defined as cell fragments. Apoptotic fractions were calculated as R2/(R1 + R2). The percentages of cell fragments were calculated as R3/(R1+R2+R3). The lines on the contour plots represent cell numbers increasing with 40% between each level cytometer from regions R2 (Figure 1B) were air dried on object glasses and covered by 8 μl of antifade. They were then observed in a Zeiss axioplan fluorescence microscope equipped with a cooled CCD camera (CCD 3200, Astromed, Cambridge, UK) that was operated by an image processing program (Astromed/ Visilog). A HBO/100-W mercury lamp was used for excitation. The microscope was equipped with a 470–490 nm bandpass excitation filter and a 500-nm dichroic beam splitter. The fluorescence was imaged through a 520–550 nm bandpass filter.

### RESULTS

In the present work, we have compared the type of cell death and its kinetics elicited by PDT-induced damage to different intracellular sites. V79 cells were killed by PDT with four dyes with different physicochemical properties. Two of the dyes, the hydrophilic $TPPS_4$ and the more lipophilic $TPPS_{2a}$, are taken up into the lysosomes of V79 cells by endocytosis and are redistributed to the cytosol ($TPPS_4$) and the endoplasmic reticulum ($TPPS_{2a}$) after light exposure (Berg et al, 1991; Rodal et al, 1998). The other two dyes, 3THPP and Photofrin, which are more lipophilic, localize in intracellular membranes but do not seem to have any preference for lysosomal membranes (Berg and Moan, 1994).

### Identification of apoptotic cells by flow cytometry

Figure 1 shows examples of flow cytometric data for three photosensitizers to illustrate how apoptotic cells were distinguished. Gates were positioned on contour plots for PDT-treated control samples that had been fluorescence stained with streptavidin–fluorescein in the absence of TdT, that is without the enzyme that specifically elongates DNA strand breaks (Figure 1A), as described in Materials and methods. The fluorescence in these samples represents the background staining of the cells. On plots of TdT-treated samples (Figure 1B), cells with background staining and without specifically stained DNA strand breaks are found in regions R1, whereas cells with an increased number of specifically stained DNA strand breaks compared with the background level appear in regions R2. Regions R3 include signals from cell fragments with a DNA content of less than half that in $G_1$ cells. This was controlled for by fluorescence (Figure 2) and electron microscopy (not shown), which shows that the cells in regions R2 also had the typical apoptotic morphology.

© Cancer Research Campaign 1999

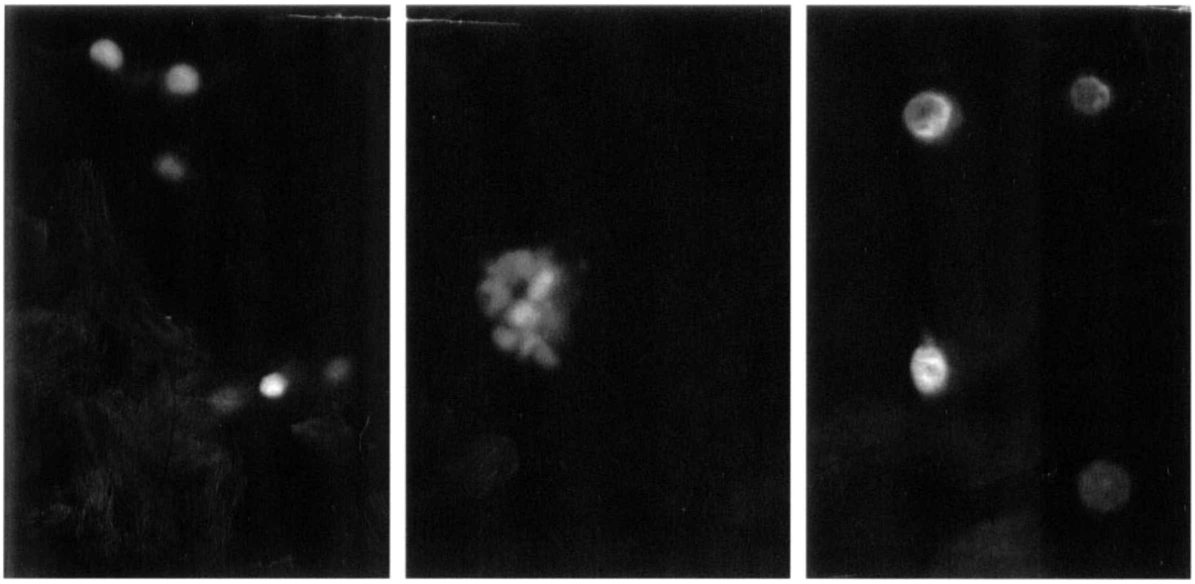

**Figure 2** Fluorescence micrographs of specifically stained DNA strand breaks in the nuclei of apoptotic cells obtained 24 h after PDT treatment with $TPPS_4$, $TPPS_{2a}$ or 3THPP. The pictures show six ($TPPS_4$), one ($TPPS_{2a}$) and four (3THPP) representative apoptotic nuclei respectively. The scale is the same for all cells. Control V79 cells showed only a weak background staining of the whole cells significantly lower than the staining of the nuclei of apoptotic cells (not shown)

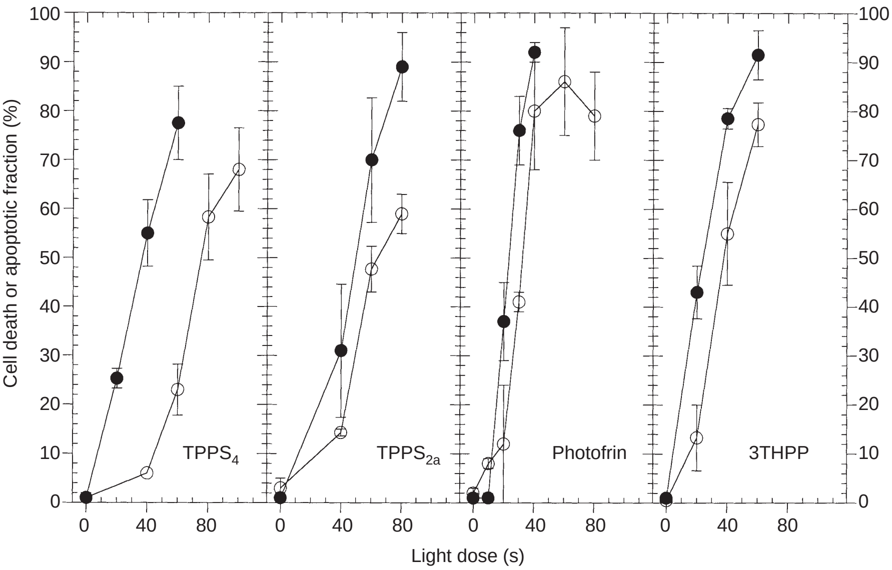

**Figure 3** Total cell death (●) and apoptosis (○) induced in V79 cells that were PDT-treated with different photosensitizers and increasing light doses. The cells were incubated with $TPPS_4$ (180 μg $ml^{-1}$), $TPPS_{2a}$ (0.5 μg $ml^{-1}$), Photofrin (2.5 μg $ml^{-1}$) or 3THPP (0.2 μg $ml^{-1}$) for 18 h until irradiation. Total cell death was measured 26 h after PDT in a cell survival assay, as described in Materials and methods. The apoptotic fractions were obtained from flow cytometric measurements on cells fixed 27 h after irradiation, as described in Figure 1 and Materials and methods. Bars represent the range for 3–6 experiments

### Induction of apoptosis with PDT

In Figure 3, the apoptotic fractions measured 27 h after PDT by flow cytometry are plotted in a light dose-dependent manner. In addition, total cell death, measured 26 h after PDT in a cell survival assay, is plotted. The figure shows that all dyes tested did induce apoptosis in V79 cells but to a different extent. Also, a fifth tetraphenylporphyrin, the positively charged $TMPyPH_2$ which is taken up into lysosomes and redistributed to the cytosol after PDT, did induce apoptosis (data not shown). To study the importance of dye aggregates on the cell surface, in some experiments the dye-incubated cells were brought to sensitizer-free medium during the last hour before irradiation. Figure 4 shows that the three dyes tested in combination with light still induced apoptosis. For the most lipophilic dye, 3THPP, total cell death and induction of apoptosis were influenced only to a minor extent by the washing procedure. For $TPPS_4$ and $TPPS_{2a}$, both the apoptotic fractions and total cell death were reduced by a factor of approximately 2.

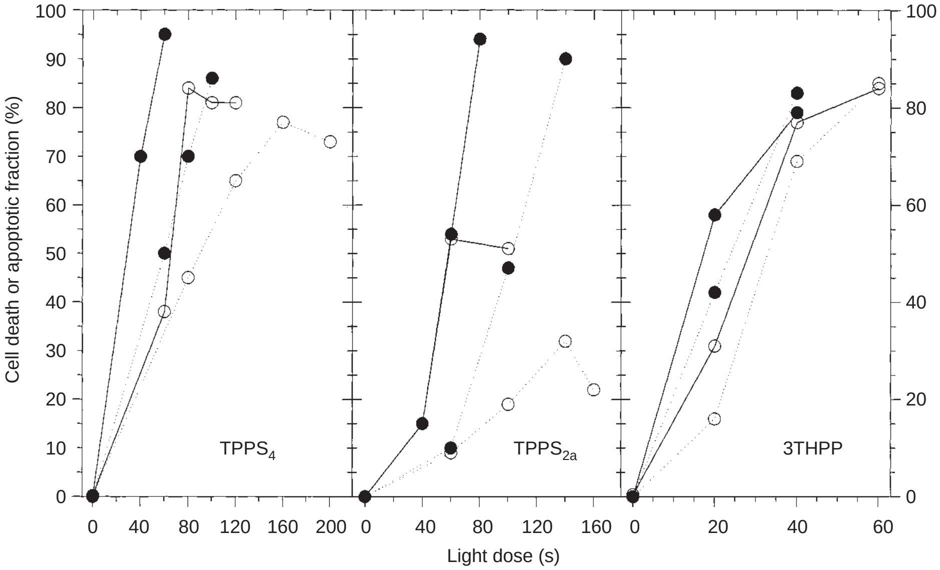

**Figure 4** The effect on total cell death (●) and on the apoptotic fractions (○) of incubating the dye-containing cells in photosensitizer-free medium during 1 h before light activation. Data are shown for $TPPS_4$, $TPPS_{2a}$ and 3THPP relative to increasing light doses. One representative experiment is shown including parallels for washed (....) and unwashed (——) cells. The cells were treated with PDT as described in Figure 3

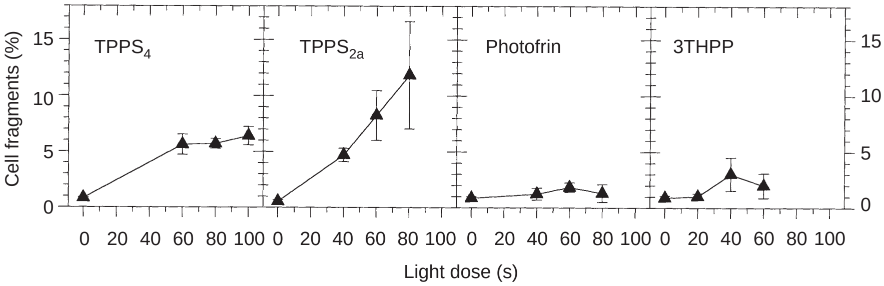

**Figure 5** The percentage of cell fragmentation measured at 27 h after PDT is shown for $TPPS_4$, $TPPS_{2a}$, Photofrin and 3THPP relative to the light dose applied. Fragmentation was quantified as described in Figure 1. Bars represent the range for three or four experiments

### Quantification of PDT-induced apoptosis initiated at different localizations

Quantification of the apoptotic fractions by flow cytometry, as described in Materials and methods, indicated that the dyes induced apoptosis to a varying extent compared with total cell death (Figure 3). For the lipophilic 3THPP and Photofrin, the curves for total cell death and apoptosis seemed to overlap within the limits of the method. This was not the case with the more hydrophilic $TPPS_4$ and $TPPS_{2a}$. A relatively lower apoptotic fraction was found and may be explained by extensive disintegration of part of the cells. Thus, loss of cells from the PDT-treated samples before flow cytometric measurements was controlled by counting the cell numbers in a Coulter counter. Both loose cells in the medium and cells scraped off the bottom of the cell culture flasks were included. Cell numbers were counted in two parallel samples before irradiation and at 24 h after PDT doses killing about 90% of the cells. Comparison of the cell numbers obtained from untreated V79 cells that were scraped off and trypsinized, respectively, showed that about one third of the cells were lost because of scraping the cells off. However, this was our method of choice because apoptotic cells were sensitive to trypsinization. Among the PDT-treated cells, the cell numbers obtained 1 day after PDT treatment seemed to remain constant for all dyes except for $TPPS_4$. In this case, another 65% of the cells were lost from the samples.

### Cell fragmentation

The fact that apoptotic fractions were smaller than the total number of cell deaths can also be due to formation of cells or cell fragments with a reduced DNA content that are gated in regions R3 of the flow cytometric plots (see Figure 1A). The measured percentage of cell fragments is presented for the four dyes in a light dose-dependent manner in Figure 5. The curves show clearly that none or only a few cell fragments appeared after PDT with the membrane-localized lipophilic dyes Photofrin and 3THPP. On the contrary, among cells PDT-treated with $TPPS_{2a}$ and $TPPS_4$, cell fragmentation increased with the light dose.

Cell fragmentation can be due to necrosis or apoptotic body formation. Apoptotic bodies are thought to contain more DNA strand breaks than living cells. In Figure 6, histograms are presented showing the numbers of cell fragments gated in regions R3 of the flow diagrams (Figure 1B) relative to the intensity of green fluorescence representing the amount of DNA strand breaks at different time stages after PDT. Two waves of cell fragmentation seemed to occur. The first contained cell fragments with a low

 © Cancer Research Campaign 1999

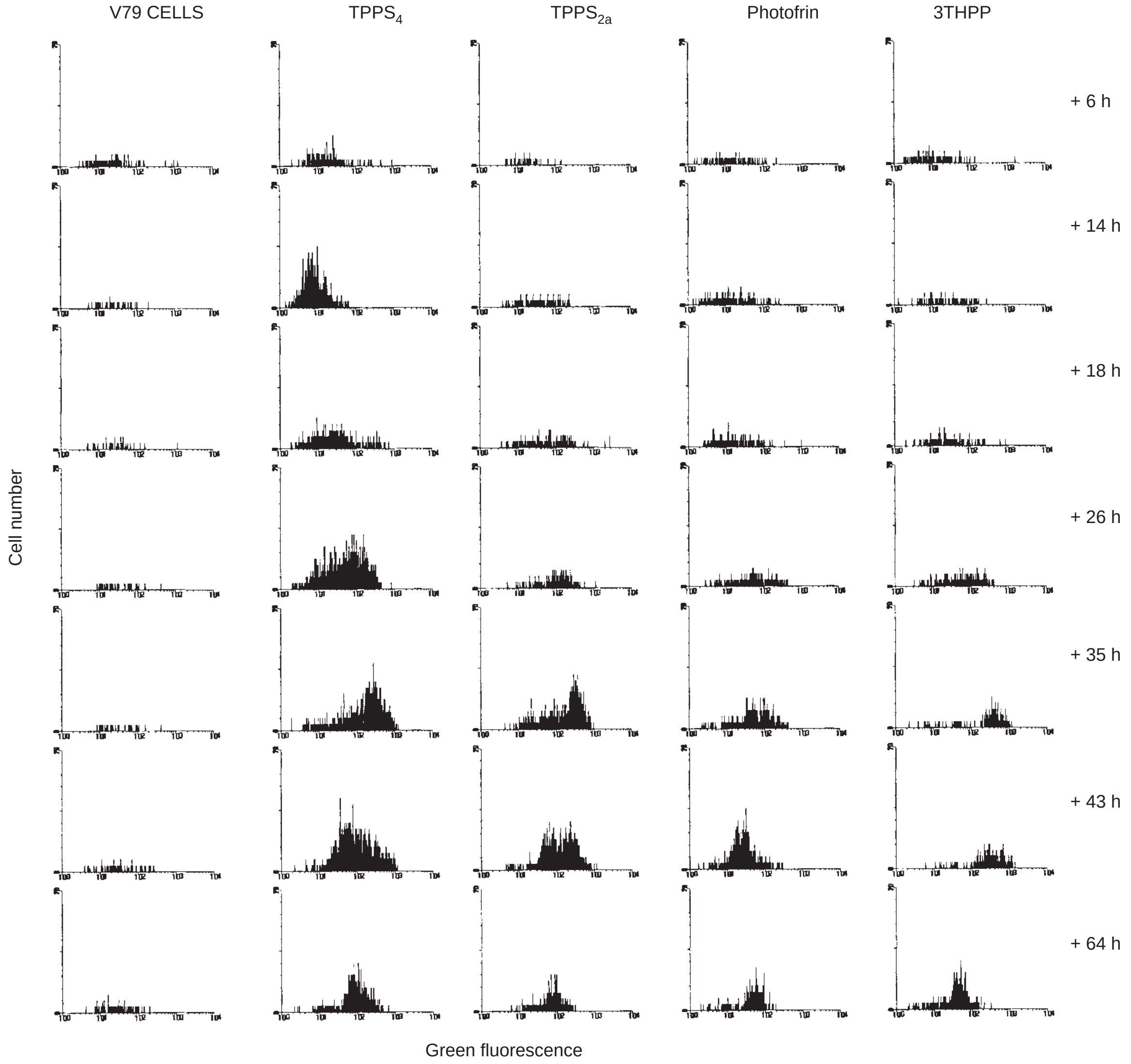

**Figure 6** Distribution of DNA strand breaks in TdT-stained cell fragments (gated from regions R3, see Figure 1B) appearing at different time intervals after PDT with $TPPS_4$, $TPPS_{2a}$, Photofrin and 3THPP or in untreated V79 cells. The green fluorescence, here with an intensity less than the absolute number 40 is due to background staining (see Figure 1). Larger values represent an increased number of DNA strand breaks in the cell fragments. The data are measured in $10^4$ cells for all dyes in parallel in one representative experiment content of DNA strand breaks which seemed to disappear within the first 26 h after PDT. At about that time point, a second wave appeared of cell fragments with a higher content of DNA strand breaks. Both the number of cell fragments and their content of DNA strand breaks increased until they reached a maximum at about 35 h after PDT when both parameters again decreased. For the more lipophilic dyes, Photofrin and 3THPP, both waves were relatively small. For the more hydrophilic dye, $TPPS_{2a}$, the second wave of cell fragments containing higher DNA strand break levels seemed to be larger and comprised a higher number of cell fragments, as confirmed by Figures 5 and 7. For the most hydrophilic dye, $TPPS_4$, both waves of cell fragmentation were pronounced.

### Morphology of the apoptotic nuclei

The morphology of the apoptotic nuclei was studied by fluorescence microscopy after sorting out apoptotic cells from regions R2 with the flow cytometer. Fluorescence micrographs are shown for $TPPS_4$, $TPPS_{2a}$ and 3THPP at 24 h after PDT (Figure 2). Control V79 cells showed only background staining significantly weaker than the staining of apoptotic nuclei (therefore not shown). In apoptotic nuclei, the fluorescence from the stained DNA strand breaks overlapped with that from Hoechst 33258-stained total DNA and the micrographs looked identical. The fluorescence-stained DNA of 3THPP-treated cells confirmed the traditional view of early apoptotic nuclei (Kerr et al, 1972) with condensed chromatin in the nuclear periphery. In $TPPS_{2a}$–PDT-treated cells, the nuclei had fragmented within the cells. With $TPPS_4$, the fluorescence was concentrated in one spot per cell instead of forming a cap or several separate spots.

### The kinetics of apoptosis induced by PDT

Figure 8 shows the kinetics of PDT-induced apoptosis over the time period of 63 h. Among control cells, the apoptotic fraction

*© Cancer Research Campaign 1999*

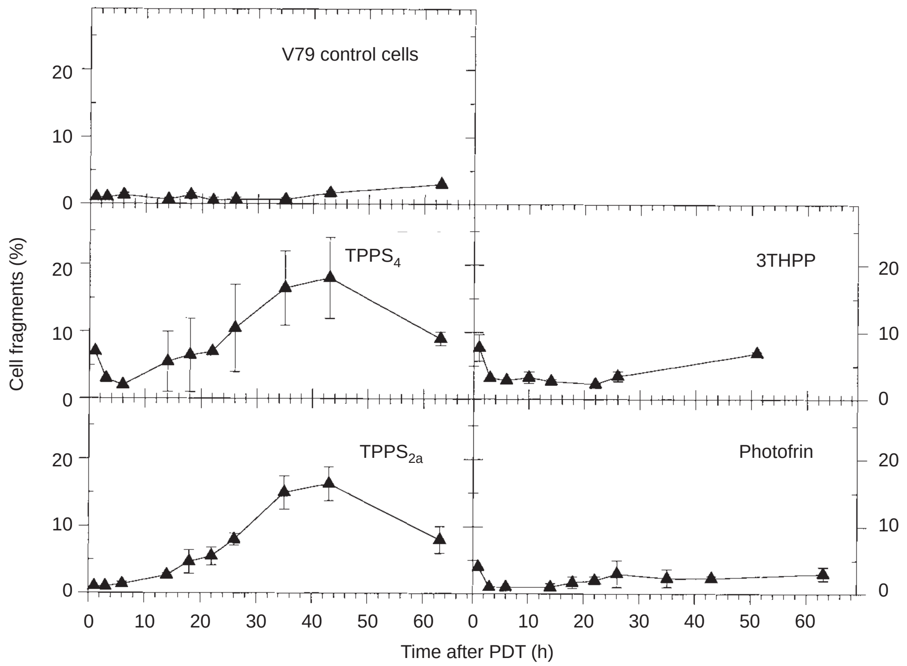

**Figure 7** The time dependence of the percentage of cell fragments after PDT with $TPPS_4$, $TPPS_{2a}$, Photofrin, 3THPP as well as for untreated control cells. Cell fragmentation was measured by flow cytometry (see Figure 1) in parallel with the apoptotic fractions in Figure 8. Bars show the range for two or three experiments

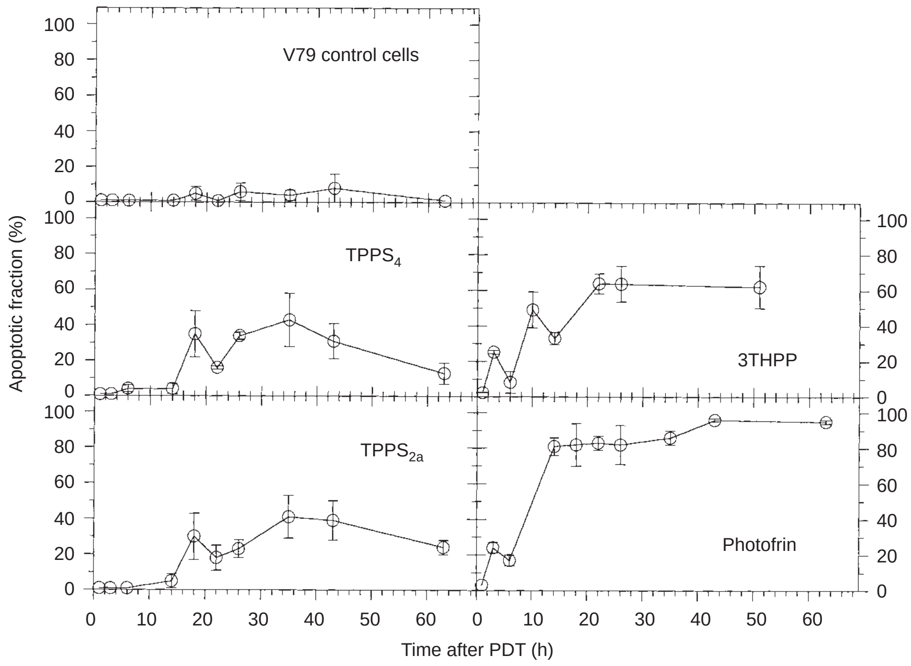

**Figure 8** The time dependence of the apoptotic fractions induced by PDT with different photosensitizers in V79 cells. The cells were treated with PDT doses corresponding to 80% ($TPPS_4$, 3THPP) and 95% total cell death ($TPPS_{2a}$, Photofrin). Untreated V79 cells are included as a control. The samples were stained by the TdT assay as described in Materials and methods and the apoptotic fractions were calculated from flow cytometric measurements as described in Figure 1. The bars represent the range for three experiments (two for 3THPP)

was less than 10% throughout the whole time course. The membrane-localized, lipophilic dyes Photofrin and 3THPP induced an immediate response within 3 h after PDT. The apoptotic fractions reached the level of total cell death, as measured in the cell survival assay (Figure 3), after about 14–22 h. They remained stable at this high level and did not decrease. In samples PDT-treated with $TPPS_{2a}$ or $TPPS_4$, apoptotic cells appeared increasingly from 18 h after PDT. The apoptotic fractions did not reach the level of total cell death measured in the cell survival assay and declined from about 35 h after PDT treatment.

 *© Cancer Research Campaign 1999*

### The kinetics of cell fragmentation

Cell fragmentation as a function of time after PDT is shown in Figure 7. Some cell fragmentation was observed immediately after PDT with all dyes. Apart from that, the lipophilic dyes and light induced fragmentation only to a minor extent at a late stage. The more hydrophilic dyes $TPPS_{2a}$ and $TPPS_4$ induced cell fragmentation to a high extent, as seen in Figures 5 and 6. Cell fragmentation reached a maximum level at about 43 h after PDT. The relative number of cell fragments then decreased again, probably because of secondary necrosis or loss of small cell fragments resulting from ongoing fragmentation. The time lag between the maxima of apoptotic fractions and that of cell fragmentation was about 8 h.

### DISCUSSION

PDT-induced apoptosis has been shown for a number of different cell types, both in vitro (Agarwal et al, 1991; He et al, 1994; Luo et al, 1996; Noodt et al, 1996) and in vivo (Zaidi et al, 1993; Agarwal et al, 1996; Webber et al, 1996). Induction of apoptosis has been claimed to be both cell type specific and sensitizer dependent (Luo et al, 1996). In the present study, three tetraphenylporphyrins and Photofrin with varying lipophilicity and intracellular localization were compared with respect to their potential to induce apoptosis in V79 cells in combination with light. Apoptotic cells could be detected by flow cytometry after specific staining of DNA strand breaks in samples of PDT-treated cells treated with both lipophilic dyes localized in intracellular membranes (3THPP and Photofrin) and more hydrophilic dyes taken up into lysosomes by the endocytic pathway ($TPPS_{2a}$ and $TPPS_4$) (Figures 3 and 8). A fifth tetraphenylporphyrin, the positively charged $TMPyPH_2$, also induced apoptosis (data not shown). Previously, we found apoptotic features in V79 cells after PDT treatment with a new photosensitizer, methylene blue derivative (Noodt et al, 1998), and with 5-aminolaevulinic acid (ALA)-induced protoporphyrin IX (Noodt et al, 1996). It was confirmed by electron microscopy that the ALA-induced apoptotic cells detected by flow cytometry had the typical morphology of apoptotic cells and that the characteristic ladder of DNA fragments could be isolated by gel electrophoresis (Noodt et al, 1996). In the present study, apoptotic morphology was confirmed by light, electron (data not shown) and fluorescence microscopy (Figure 2).

PDT with all dyes tested in the present work induced apoptosis, but different pathways seemed to be engaged. While the membrane-localized dyes, Photofrin and 3THPP, induced DNA strand-break formation immediately after PDT, the other two dyes, $TPPS_{2a}$ and $TPPS_4$, did so increasingly from 18 h after PDT (Figure 8). In the literature, the collapse of the inner mitochondrial membrane potential is now considered as one possible crucial event for rapid induction of apoptosis (Kroemer, 1997) sufficient to provoke the entire range of apoptosis-associated events, including DNA fragmentation (Kroemer et al, 1997). The transition of the mitochondrial membrane potential and release of apoptogenic factors such as apoptosis-inducing factor (AIF) (Susin et al, 1996) is under regulation of Bcl-2-related proteins that are localized in membranes of mitochondria, the endoplasmic reticulum and the nuclear envelope (Krajewski et al, 1993). This correlates with the intracellular distribution of the lipophilic photosensitizers and Bcl-2 or a related protein is probably involved in rapid induction of apoptosis by PDT. Other photosensitizers localized in mitochondrial membranes, such as methylene blue derivative and ALA-induced protoporphyrin IX, have also been shown to induce apoptosis rapidly within 3–4 h after PDT (Noodt et al, 1996, 1998). Recently, a photosensitizer localized in the endoplasmic reticulum has been shown to induce activation of caspase-3 followed by PARP cleavage and DNA fragmentation (Granville et al, 1997), whereas Bcl-2 overexpression has been shown to increase resistance to PDT-induced apoptosis in a cell line (He et al, 1996).

Another striking finding from the present data was that different apoptotic features seemed to be induced by PDT with the various dyes, dependent on the site of initial damage. Although DNA was readily fragmented after PDT with the membrane-localized dyes Photofrin and 3THPP, none or only a few cells with a reduced DNA content (Figures 5 and 7) appeared in regions R3 of the flow diagrams (see Figure 1), and no nuclear fragmentation could be detected by fluorescence microscopy (Figure 2). The fraction of apoptotic cells reached the level of total cell death and the apoptotic cells stayed present in the samples during the rest of the experiments (Figure 8). Lazebnik et al (1995) have shown that lamins, the constituents of the nuclear lamina, are cleaved by an interleukin-1β-converting enzyme-related protease (caspase). This enzymatic activity can be inhibited by 'tosyllysine chloromethyl ketone' (TCLK). Nuclear fragmentation and formation of apoptotic bodies were prevented by TCLK but not DNA fragmentation and chromatin condensation. Correspondingly, PDT with the lipophilic dyes may have a similar effect as TCLK or a stabilizing effect on the nuclear lamina, e.g. by cross-linking between amino acids or between amino acids and DNA (Dubbelman et al, 1982).

In contrast, the more hydrophilic lysosome-localized dyes $TPPS_{2a}$ and $TPPS_4$ induced cell fragmentation in combination with light, as shown in Figures 5, 6 and 7. Interestingly, only $TPPS_{2a}$ induced nuclear fragmentation (Figure 2), whereas the nuclei of cells after $TPPS_4$-PDT had a pyknotic appearance, as described previously (Kerr et al, 1972), indicating that a process other than fragmentation is responsible for the nuclear changes. Loss of fragmented DNA from apoptotic cells that become permeabilized at a late stage has been described by others (Darzynkiewicz et al, 1992), and in the present work apoptotic cells were shown to become increasingly permeable to propidium iodide 24 h after $TPPS_4$-PDT. Differences in the fragmentation process may be related to the localization of the dyes in the cytosol ($TPPS_4$) and endoplasmic reticulum ($TPPS_{2a}$) after light-induced release from the lysosomes (Berg et al, 1991; Rodal et al, 1998). The present results may provide evidence that degradative events in apoptosis are performed by distinct executors that can be activated independently of each other (Lazebnik et al, 1995; Takahashi et al, 1997a, 1997b).

Not only the pathway for induction of apoptosis and the set of induced apoptotic features seemed to depend on the site of initial damage. Also, the relative number of apoptotic cells varied compared with necrosis. In the case of $TPPS_4$, several findings indicate necrotic cell death in addition to apoptotic body formation. Figure 6 shows that a prominent peak of TdT-negative cell fragments appeared 14 h after PDT. A substantial loss of cells after $TPPS_4$-PDT during the staining procedures of the TdT assay was measured by Coulter counting and is described in the Results section. Propidium iodide staining of apoptotic cells induced by $TPPS_4$-PDT as described above showed that the plasma membrane integrity was lost increasingly after 24 h, indicating secondary necrosis.

The problems connected to quantification of apoptotic cells have been addressed in a more general context by others (Potten, 1996). Our data provide examples of some of the obstacles that

© Cancer Research Campaign 1999

exist to the identification and quantification of apoptotic cells. Apoptotic cells are often assessed and quantified by detection of a subdiploid top on DNA histograms measured by flow cytometry (Darzynkiewicz et al, 1992). The most efficient inducers of apoptosis in this study, 3THPP and Photofrin, induced DNA fragmentation, but no subdiploid top appeared on the DNA histograms. The time window for measurement of maximal values of DNA or cell fragmentation varied with the dye applied, even within the same cell type (Figures 7 and 8 and Noodt et al, 1996). Cell fragmentation provides another obstacle to quantification of apoptosis. Necrotic cell fragments are easily lost during the staining procedures, such that apoptotic cells are quantified relative only to the remaining intact and apoptotic cells and overestimated. Apoptotic body formation results in underestimation of the apoptotic fraction because fragmented apoptotic cells are not taken into account. In our study, quantification was further complicated because of incomplete separation of the apoptotic cells in the fluorescence-staining assay (see Figure 1B). The gates were placed on plots for control samples with background staining only (Figure 1A; –TdT), such that regions R1 contained 98% of the signals. On the plots for the specifically stained samples (Figure 1B), the cells appearing in regions R1 were defined as non-apoptotic, while apoptotic cells were counted from regions R2. This method was tested in numerous measurements and gave reproducible results that correlated with the cell survival measurements. The morphology of the cells gated in regions R2 was confirmed as being apoptotic by phase contrast and fluorescence microscopy after sorting the cells by the FACStar flow cytometer (Figure 2).

In conclusion, our results indicate that PDT induces apoptosis in V79 cells via different pathways and to a varying extent is dependent on the initial site of damage. Furthermore, different features of apoptosis are induced. The exact localizations from where each single event is triggered have to be studied further. A knowledge of these sites could give clues to which triggers and intracellular messengers involved in PDT-induced apoptosis.

### ACKNOWLEDGEMENTS

We thank Torstein Schjerven and Kirsti Solberg for expert assistance on data technology handling and flow cytometry respectively. This work was supported by the Norwegian Radium Hospital, the Research Council of Norway and the Norwegian Cancer Society.

### ABBREVIATIONS

V79 cells, Chinese hamster lung fibroblasts; PDT, photodynamic therapy; $TPPS_n$, *n*-sulphonated *meso*-tetraphenylporphine; 3THPP, tetra(3-hydroxyphenyl)porphyrin; TdT, terminal deoxynucleotidyl transferase.

### REFERENCES

Agarwal ML, Clay ME, Harvey EJ, Evans HH, Antunez AR and Oleinick NL (1991) Photodynamic therapy induces rapid cell death by apotosis in L5178Y mouse lymphoma cells. *Cancer Res* **51**: 5993–5996

Agarwal R, Korman NJ, Mohan RR, Feyes DK, Jawed S, Zaim MT and Mukhtar H (1996) Apoptosis is an early event during phtalocyanine photodynamic therapy-induced ablation of chemically induced squamous papillomas in mouse skin. *Photochem Photobiol* **63**: 547–552

Berg K (1996). Mechanisms of cell damage in photodynamic therapy. In *The Fundamental Bases of Phototherapy*, Honigsmann H, Jori G and Young A (eds.), pp. 181–207. OEMF: Milano

Berg K and Moan J (1994) Lysosomes as photochemical targets. *Int J Cancer* **59**: 814–822

Berg K, Western A, Bommer JC and Moan J (1990) Intracellular localization of sulfonated *meso*-tetraphenylporphines in a human carcinoma cell line. *Photochem Photobiol* **52**: 481–487

Berg K, Madslien K, Bommer JC, Oftebro R, Winkelman JW and Moan J (1991) Light induced relocalization of sulfonated *meso*-tetraphenylporphines in NHIK 3025 cells and effects of dose fractionation. *Photochem Photobiol* **53**: 203–210

Darzynkiewicz Z, Bruno S, Del Bino G, Gorczyca W, Hotz MA, Lassota P and Traganos F (1992) Features of apoptotic cells measured by flow cytometry. *Cytometry* **13**: 795–808

Dubbelman TMAR, Van Steveninck AL and Van Steveninck J (1982) Hematoporphyrin-induced photooxydation and photodynamic cross-linking of nucleic acids and their constituents. *Biochem Biophys Acta* **719**: 47–52

Fisher DE (1994) Apoptosis in cancer therapy: crossing the threshold. *Cell* **78**: 539–542

Gorczyca W, Gong J and Darzynkiewicz Z (1993) Detection of DNA strand breaks in individual apoptotic cells by the in situ terminal deoxynucleotidyl transferase and nick translation assays. *Cancer Res* **53**: 1945–1951

Granville DJ, Levy JG and Hunt DWC (1997) Photodynamic therapy induces caspase-3 activation in HL-60 cells. *Cell Death Differ* **4**: 623–628

He X-Y, Sikes RA, Thomsen S, Chung LWK and Jaques SL (1994) Photodynamic therapy with Photofrin II induces programmed cell death in carcinoma cell lines. *Photochem Photobiol* **59**: 468–473

He J, Agarwal ML, Larkin HE, Friedman LR, Xue L-Y and Oleinick NL (1996) The induction of partial resistance to photodynamic therapy by the protooncogene bcl-2. *Photochem Photobiol* **64**: 845–852

Henderson BW and Dougherty TJ (1992) How does Photodynamic therapy work? *Photochem Photobiol* **55**: 145–157

Hickman JA (1996) Apoptosis and chemotherapy resistance. *Eur J Cancer* **32**: 921–926

Kerr JFR, Wyllie AH and Currie AR (1972). Apoptosis: a basic biological phenomenon with wide-ranging implications in tissue kinetics. *Br J Cancer* **26**: 239–257

Kerr JFR, Winterford CM and Harmon BV (1994) Apoptosis – its significance in cancer and cancer therapy. *Cancer* **73**: 2013–2026

Krajewski S, Tanaka S, Takayama S, Schibler MJ, Fenton W and Reed JC (1993) Investigation of the subcellular distribution of bcl-2 oncoprotein: residence in the nuclear envelope, endoplasmic reticulum and outer mitochondrial membranes. *Cancer Res* **53**: 4701–4714

Kroemer G (1997) The proto-oncogene Bcl-2 and its role in regulating apoptosis. *Nature Med* **3**: 614–620

Kroemer G, Zamzami N and Susin SA (1997) Mitochondrial control of apoptosis. *Immunol Today* **18**: 44–51

Lazebnik YA, Takahashi A, Moir RD, Goldman RD, Poirier GG, Kaufmann SH and Earnshaw WC (1995) Studies of the lamin proteinase reveal multiple parallel biochemical pathways during apoptotic execution. *Proc Natl Acad Sci USA* **92**: 9042–9046

Luo Y and Kessel D (1997) Initiation of apoptosis versus necrosis by photodynamic therapy with chloroaluminum phtalocyanine. *Photochem Photobiol* **66**: 479–483

Luo Y, Chang CK and Kessel D (1996) Rapid induction of apoptosis by photodynamic therapy. *Photochem Photobiol* **63**: 528–534

Moan J and Berg K (1991) The photodegradation of porphyrins in cells can be used to estimate the lifetime of singlet oxygen. *Photochem Photobiol* **53**: 549–553

Noodt BB, Kvam E, Steen HB and Moan J (1993) Primary DNA damage, HPRT mutation and cell inactivation photoinduced with various sensitizers in V79 cells. *Photochem Photobiol* **58**: 541–547

Noodt BB, Berg K, Stokke T, Peng Q and Nesland JM (1996) Apoptosis and necrosis induced with light and 5-aminolevulinic acid-derived protoporphyrin IX. *Br J Cancer* **74**: 22–29

Noodt BB, Rodal GH, Wainwright M, Peng Q, Horobin R, Nesland JM and Berg K (1998) Apoptosis induction by different pathways with methylene blue derivative and light from mitochondrial sites in V79 cells. *Int J Cancer* **75**: 941–948

Potten CS (1996) What is an apoptotic index measuring? A commentary. *Br J Cancer* **74**: 1743–1748

Rodal GH, Rodal SK, Moan J and Berg K (1998) Liposome-bound Zn (II) phthalocyamine. Mechanisms for cellular uptake and photosensitization (submitted).

Soini Y, Virkajärvi N, Lehto V-P and Pääkkö P (1996) Hepatocellular carcinomas with a high proliferation index and a low degree of apoptosis and necrosis are associated with a shortened survival. *Br J Cancer* **73**: 1025–1030

 © Cancer Research Campaign 1999

Susin SA, Zamzami N, Castedo M, Hirsch T, Marchetti P, Macho A, Daugas E, Geuskens M and Kroemer G (1996) Bcl-2 inhibits the mitochondrial release of an apoptogenic protease. *J Exp Med* **184**: 1331–1341

Takahashi A, Goldschmidt-Clermont PJ, Alnemri ES, Fernandes-Alnemri T, Yoshizawa-Kumagaya K, Nakajima K, Sasada M, Poirier, GG and Earnshaw WC (1997*a*). Inhibition of ICE-related proteases (Caspases) and nuclear apoptosis by phenylarsine oxide. *Exp Cell Res* **231**: 123–131

Takahashi A, Hirata H, Yonehara S, Imai Y, Lee K-K, Moyer RW, Turner PC, Mesner PW, Okazaki T, Sawai H, Kishi S, Yamamoto K, Okuma M and Sasada M (1997*b*). Affinity labeling displays the stepwise activation of ICE-related proteases by FAS, staurosporine and CrmA-sensitive caspase-8. *Oncogene* **14**: 2741–2752

Webber J, Luo Y, Crilly R, Fromm D and Kessel D (1996) An apoptotic response to photodynamic therapy with endogenous protoporphyrin in vivo. *J Photochem Photobiol B*:*Biol* **35**: 209–211

Weishaupt KR, Gomer CJ and Dougherty TJ (1976) Identification of singlet oxygen as the cytotoxic agent in photo-inactivation of a murine tumor. *Cancer Res* **36**: 2326–2329

Wyllie AH, Kerr JFR and Currie AR (1980) Cell death: the significance of apoptosis. *Int Rev Cytol* **68**: 251–306

Zaidi SIA, Oleinick NL, Zaim MT and Mukhtar H (1993) Apoptosis during photodynamic therapy-induced ablation of RIF-1 tumors in C3H mice: electron microscopic, histopathologic and biochemical evidence. *Photochem Photobiol* **58**: 771–776

Zhou C, Shunji C, Jinsheng D, Junlin L, Jori G and Milanesi C (1996) Apoptosis of mouse MS-2 fibrosarcoma cells induced by photodynamic therapy with Zn (II)-phtalocyanine. *J Photochem Photobiol B*:*Biol* **33**: 219–223

*© Cancer Research Campaign 1999*
